# New York Odontological Society

**Published:** 1891-06

**Authors:** 

**Affiliations:** New York Odontological Society


### Reports of Society Meetings.

### NEW YORK ODONTOLOGICAL SOCIETY.

The regular monthly meeting of the New York Odontological Society was held Tuesday morning, March 17, 1891, at eight o'clock, at the New York Academy of Medicine, No. 17 West Forty-third Street.

The President, Dr. W. H. Dwinelle, in the chair.

### INCIDENTS OF OFFICE PRACTICE AND CASUAL COMMUNICATIONS.

*Dr. A. H. Brockway.*—I would like to relate a little incident of practice, called to mind by reference, at the last meeting, in Dr. Rollins's paper, to galvanic action in the mouth, from the proximity of fillings of different materials. The incident is as follows:

A lady patient came to me, some months ago, for an appointment, complaining that she had very singular sensations in her mouth, which she attributed to galvanic action. She is a woman of remarkable intelligence, and I was somewhat impressed by her statement. Endeavoring to ascertain the cause of the galvanic action complained of, I discovered two newly-formed cavities in the proximate surfaces of adjoining bicuspid teeth, rather obscure cavities, and at once suspected that the cause of her sensations was there. I filled those cavities and it put an end to the galvanic action of which she complained. I question if disturbing galvanic action from the presence of differing filling materials is as common as some suppose. I am not sure that I have ever seen a case of it except from the contact of different fillings in opposing teeth when the jaws are brought together.

*Dr. Van Woert.*—I have with me a patient whom I presented to Dr. Brockway while at my office last Sunday; and it was at his suggestion that the case is brought here for your inspection. The patient, a gentleman, presented himself to me about a year ago, with a fairly good-sized fissure in the soft palate, which had been acquired within a few weeks, and had rendered the articulation of sound so imperfect that it was almost impossible to understand anything he said. I had not seen the patient since, until Sunday last, when he again presented himself. The fissure had closed, yet the articulation of sound has not been restored, and it is with great difficulty that the gentleman is able to converse.

I had no difficulty in diagnosing the case as one of syphilis, but I am free to confess that I am not conversant enough with such difficulties to determine where the defects are which cause the imperfect articulation. If it is your pleasure, Mr. President, I shall be glad to exhibit the patient, and the gentlemen may draw their own conclusions.

*Dr. S. G. Perry.*—In reference to Dr. Brockway's statement, I think the inference would be that he thinks galvanic action never occurs. I believe that it does not often occur, but I should hesitate about accepting the idea that it never occurs. I have seen a few instances of it caused by the contact of different metals in different teeth, but never, I think, from different metals in the same tooth. A connecting chain of fillings in the upper and lower jaw, I think, can sometimes, when of different metals, cause galvanic disturbance. In reference to this case shown by Dr. Van Woert, it seems remarkable that there should have been a spontaneous closing of the fissure. I think that Dr. Kingsley has always claimed that but little improvement is to be expected in such a case until the voice and speech of the patient have been educated. This gentleman will speak better, I think, when he has used certain methods of educating the voice; as yet he has not acquired the art of speaking with the mouth in that new condition. I am not, however, an authority on these subjects. It is a very curious and interesting case.

*Dr. Brockway.*—I did not mean to be understood as saying that galvanic action never occurred. As Artemus Ward used to say, it "rarely seldom" occurs. The point I wished to make was that even very intelligent patients get absurd notions into their heads, and it requires some conclusive proof to convince them that they have not a galvanic battery in their mouths, which is working to their serious injury. I know very well that galvanic action does occur from the contact of fillings of different metals, but the danger from it is vastly less than is usually supposed.

*The President.*—This case presented by Dr. Van Woert is certainly very interesting. I regret that Dr. Kingsley is not with us, for he is authority on these matters.

I see a number of new members present to-night, and I feel as though it were proper to say a few words to them. I congratulate you, gentlemen, and ourselves, upon your appearance with us, and hope you will continue to meet with us on all occasions. We extend the right hand of fellowship to you, and welcome you to our association, expecting you to take part with us in all duties and deliberations. We have need of you, as, in the order of nature, we, the older members, will pass away, and you are to take our places. It is fitting, then, that you should be preparing the way for your future responsibilities. There should be no falling off here; we look to the younger element to furnish the needed supply. We look to you to perpetuate our institutions and to give glory to our cause.

It will be well to cultivate, with us, the habit of speaking; no acquirement can be more valuable or useful, and we have among our members those who have no superiors in this respect. We do not know how many Miltons, Cromwells, or Washingtons may grace the future that shall have graduated from our association. You may regard this as a kind of school which may result to your advantage, as it has to many of us. We have in our midst artists and those who are *authority* in most of the departments of science; so, too, we can boast of having among our members some of the best parliamentarians of the day. Come prepared to speak, and, if you are not familiar with the subject, take notes beforehand. Remember there is a debt of gratitude we owe to the past that we can never repay. The world is entitled to our best thoughts, expressed in the best manner. Let your style be condensed, speak to the point, and briefly as compatible with your theme; do not wander from your subject, and thus increase the labors of the editor.

Come and join with us in pushing the car of progress onward.

Again we welcome you to our fraternity.

The President then introduced Dr. Heitzmann.

(For Dr. Heitzmann's paper, see page 341.)

*The President.*—Gentlemen, we have good reason to congratulate ourselves upon the privilege of listening to this very interesting address to-night, and the subject is now open for discussion. We had expected that Dr. Peirce, of Philadelphia, and Dr. Andrews, of Cambridge, would be with us, but they were prevented from coming. Dr. Atkinson is always with us, especially on an occasion like this. I hope this subject will be discussed at length to-night, for nothing can be more interesting. I will ask Dr. Atkinson to open the discussion.

*Dr. Wm. H. Atkinson.*—Mr. President and Brethren,—This is a subject that has engaged my earnest attention. By a sort of inspiration and a set of surroundings that were very favorable for investigating the formative changes in the elements of living organisms, I was engaged in their study at Cleveland, under the guidance of the then best-known histologist of that State: I need only mention Hamilton L. Smith's name as an indication of the reverence that we bore the subject and the manner of its handling.

It would be a very interesting, instructive, satisfactory, and demonstrative work to recapitulate the processes through which we were carried by the inspiration of our work,—then as novices.

Out of this organization, this protoplasm, all functioning properties are produced, as has been clearly demonstrated to-night. My heart is overflowing with gratitude and thankfulness to the great Governor of the Universe, and also to Dr. Heitzmann as the exponent of the principle, and to him all who have a mission to correct pathological conditions owe a great debt.

I wish I could have the magic power to bring every mind in this room and every member in the dental profession exactly in the stand-point I occupy myself, in regard to the beautiful work that has been presented to us to-night by the apostle of that idea. You have all heard the definition he has given to-night so clearly of what constitutes a tissue, and as to the other moot questions, he said he would not enter into a mere polemical aspect of the subject. Where is there a higher mind than his who seeks truth for her own sake, in all the fields of investigation? I have said, probably to the disgust of many, so many times, that if men had a desire to learn equal to the desire they had to be considered by their fellows learned, that we would make very much more rapid progress, that would bring us in sight of the promised land,—to show us what organism really is,—at all events, arrive at antecedents so near that we call them such.

There are some who come over here, and, individually taking up a specific line of investigation, demand that every one should bow to it, not even allowing himself to show that he understood the first principles of organization, so as to have a stand-point whereby his own conscience might be satisfied when he said yea or nay to the subjects under examination, and if, by the mercy of Heaven, correct interpretation might be had, until such progress would seize the minds of men that we would no longer be in doubt about diagnosing a case. To whom do we owe that? To my blessed old Hungarian who said that the organization depended upon living matter; unfortunately, he did not tell us what living matter was, but he said he did not know. That is worth something, If a man has an inspiration to know something that he does not yet know, it is a blessed thing that he is under the conviction that is leading him in the right direction.

Function is not understood, and it will never be understood as long as we are under the damnable pall of former teachings.

Microbes have had a black eye given them to-night by Professor Heitzmann, and I have serious doubts if there is a living example in the field of investigation that is better entitled to give us a classification than he, so that we will understand how to favor health, how to forestall disease, and thereby spread peace and good will throughout the whole realm of the sons and daughters of the Almighty, without rebuke of our own conscience, or the conscience of our fellows who are searching for the same truth we are so ardently laboring for.

Where is our difficulty? We have not yet sufficiently learned the processes through which we are carried by the grace of God in our mental work, so as to distinguish between the known and the unknown,—the unknown and the known, for we always begin with the unknown, and assume that we know it.

We have found an atom,—a thing that cannot be divided, but we never reach the truth of the hygienic movement, or a return to the health condition, by the administration of some agency which shall produce what we call the health, and what is called the disease, and what is called the remedy. Our difficulty is in assumption generally that we knew when we did not.

The doctor pronounces the fearful truth against us that for the last few years we have lost our inspiration in this work.

I wish we had the time, and you the patience, and I the inspiration, to go over that demonstration of karyokinesis, and that which has been explained to us to-night, and see that it has been acknowledged not only in the animal world, but in the vegetable world, and show that man who stands first in the discovery of protoplasm to-day,—who gave out what protoplasm was.

*Dr. John I. Hart.*—It is unnecessary, after the lucid manner in which Dr. Heitzmann has laid this matter before you, for me to corroborate what he has said; yet it is but just for me to do so, and to state that I have seen the reticulum which he has spoken of and demonstrated. At the same time, I do not think that the doctor should feel as discouraged as he seems to concerning the arguments that prevail against him, for intellectual advancement in all fields has met just such barriers as he is encountering to-day. Right through the history of development, arguments have been brought to bear against the theories that have been advanced, and in each instance, as the matter has been studied more thoroughly, admissions have been made by those who advanced the arguments against the same. The offshoots from the dental pulp were first treated as a myth, but to-day are acknowledged, and I think it is only a question of a very short time when the interconnections between these dentinal fibres will be generally admitted, just the same as the original fibres.

*Dr. J. Morgan Howe.*—I am sure we are all very much obliged to Dr. Heitzmann for his clear and graphic descriptions, but I wish to call attention to one statement that he made, which seems to imply a misapprehension. In referring to the resolution that was passed by this Society, he said that the purpose of the resolution was to *compel* himself and others to submit their specimens to the examination of experts. If I am not mistaken, the language used was carefully amended, and the resolution passed was substantially that "Drs. Heitzmann, Bödecker, and Abbott be requested to coöperate with the Society in the selection of persons to whom they would be willing to submit specimens for examination." I call attention to it merely from the fact that Dr. Heitzmann used the word "compel." The Society certainly passed the resolution with the best intent and carefully avoided objectionable suggestions. It was in the kindliest and most friendly spirit.

*Dr. Atkinson.*—I am very much pleased, and I thank Dr. Howe for the remarks he has just made. If there is any progress to be made anywhere it wants to be done in a friendly spirit, so as to avoid, if possible, any unpleasantness. It struck me, as it did Dr. Heitzmann, that it was an assumption of the exclusive ability to comprehend the thing, and that that ability resided either in the movers of the resolution or in those with whom they were cognizant. I am happy to see oil thrown on the waters, because polemical discussions have no business in scientific discussions, and it pains me to think we cannot live a good many lifetimes over to correct the mistakes we have made; and if I have anything to say to my beloved Carl Heitzmann, it is that he was always a little bit too ready to oppose the people who asserted themselves as opposed to what he saw, and I have justified him in that by reason of his greater opportunity and greater title to be regarded as competent to say that he is the best demonstrator of elementary structure of tissues, from the blood-corpuscle all the way up, that I have ever had occasion to come in contact with, and I hope that all discussions, or mere side-play, or individual preferment, to which I plead guilty, will be dropped. I always preferred those that sought the truth, and I thank him most heartily, and will give him a Delmonico dinner to show him I think so.

*Dr. Perry.*—I am exceedingly glad that the question of the resolution passed by the Society came up, and that Dr. Howe spoke of it as he did. I am sure there was a misapprehension, for it was certainly the desire of the Society to have a full and fair examination and discussion of the subject, and to do justice to Dr. Heitzmann.

While I am up I cannot help saying a word, though this subject is altogether too deep a well for me to draw water from; I may slip and fall in. In fact, it is too deep a subject for many men in any generation to undertake. It implies too much earnestness, and any one who will undertake it must devote a lifetime to it. Even if Professor Heitzmann is wrong in the theory that he holds, he has shown us by his clear statement what scientific studies will do as a school of training for the mind. It is only that training that will make men think correctly; and we ought to be ready to accept the thought of those who are so trained. There is a reasonableness about Dr. Heitzmann's theory which appeals to me very strongly.

Years ago, while studying in his laboratory, I remember distinctly that it seemed quite reasonable to consider a lump of protoplasm to be something like a bladder filled with water: compress it on one side, and it bulges out on another. Watch its movements, and one cannot help feeling that there is a boundary to it. As it contracts on one side, and the living matter becomes more concentrated, it must become thinner on the other side.

The conditions in this country are not favorable for original investigations, as Dr. Heitzmann said. I am afraid they never will be. We work too hard; life is too exacting. This is the kind of work that can never be done except by those who have the scientific spirit, and who have time at their command. I think that if Professor Miller had remained in America he would never have been the scientific investigator that he is. Professor Heitzmann, to a certain extent, takes to task his countrymen on the other side, but I think a great deal is due to them for their willingness to work so patiently for final results. We see in him a shining example of the German school.

*Dr. Remington.*—I move a vote of thanks to Dr. Heitzmann for his instructive lecture.

Motion carried.

Adjourned.

S. E. Davenport, D.D.S., M.D.S.,
*Editor New York Odontological Society.*

